# O060. Rotigotine improves drug-resistant cluster headache: a case series

**DOI:** 10.1186/1129-2377-16-S1-A106

**Published:** 2015-09-28

**Authors:** Cherubino Di Lorenzo, Gianluca Coppola, Paolo Rossi, Francesco Pierelli

**Affiliations:** 1 Don Carlo Gnocchi Onlus Foundation, Milan, Italy; 2 G.B. Bietti Foundation-IRCCS, Department of Neurophysiology of Vision and Neuro-ophthalmology, Rome, Italy; 3 Headache Clinic, INI, Grottaferrata, (RM) Italy; 4 University Consortium for Adaptive Disorders and Head pain (UCADH), Pavia, Italy; 5 Department of Medico-Surgical Sciences and Biotechnologies, Sapienza University of Rome, Latina, Italy; 6 IRCCS - Neuromed, Pozzilli, (IS) Italy

## Introduction

Cluster headache (CH) is a severe form of primary headache, characterized by trigeminal-autonomic system activation. Bouts, lasting 15-180 minutes, occur 1-8 times a day, for a period persisting for weeks or months, followed by a full remission. However, about 10-15% of patients have no remission periods and their CH is defined chronic (CCH). While symptomatic treatment is often effective, preventive treatments are limited. Our group has already reported the case of a patient affected by refractory CCH who underwent a complete and sustained response to rotigotine, a non-ergoline D3-like receptor agonist, also with a 5HT1A effect, administrated by transdermal patches. Here we report a case series of patients that have tried transdermal rotigotine for their drug-resistant CH.

## Methods

We recruited 14 CH drug-resistant patients (11 chronic) that accepted to try transdermal rotigotine to treat their CH, increasing dose weekly of 2 mg up to 6 mg, according to our previous report.

## Results

Out of 3 episodic CH (ECH) patients, two had an improvement of bouts at 4 mg, but obtained a stable benefit at 6 mg; after the presumed end of their CH period they progressively discontinued the patches and the CH did not come back till the next cluster period. The other ECH patient did not respond at 6 mg, than the dose was further increased to 8 mg, without benefit thus the treatment was interrupted. Out of 11 CCH patients, 8 were considered responders since CH disappeared (5 cases) or significantly decreased (more than 50% of reduction in terms of bouts frequency and intensity, or symptomatic consumption). Six patients had an early benefit at 4 mg but stable response at 6 mg. In two cases the dose was further increased to 8 mg since CH worsened. Two patients suddenly discontinued treatment by an ‘overnight-switch’ to pramipexole due to late dermatological reactions; one of them remained CH free after the switch. Patients who did not respond at 6 mg continued to not respond at 8 mg and treatment was interrupted.

## Discussion

This case series seems to confirm our early observation that transdermal rotigotine could act as preventive treatment in cluster headache. Further, this series give us other information: 1) Rotigotine could be increased up to 8 mg if necessary; 2) If up to 6 mg it does not act, it will not act at 8; 3) Some late dermatological complications could occur during the treatment.

Written informed consent to publication was obtained from the patient(s).

